# Supplementary material for: 16S rRNA of Mucosal Colon Microbiome and CCL2 Circulating Levels Are Potential Biomarkers in Colorectal Cancer
Source: Int J Mol Sci. 2021 Oct 4;22(19):10747. doi: 10.3390/ijms221910747 (PMC8509685; doi:10.3390/ijms221910747)
Supplement: Supplementary file 1 [file ijms-22-10747-s001.zip › ijms-1350573-Supplementary.pdf]

International Journal of
Molecular Sciences

MDPI

Supplementary Table S1. General characteristics of both the enrolled healthy control subjects (n=20, on the left) and of the CRC patients (n= 20, on the right).

Sample ID	Age (years)	Gender	BMI (Kg/m²)	Sample ID	Age (years)	Gender	BMI (Kg/m²)	Tumor location	Tumor Stage*
HC_OW/OB_01	70	F	29	CRC_01	79	F	29.4	Sigma	I
HC_OW/OB_02	67	F	37.4	CRC_02	91	F	25.78	Rectum	IIIC
HC_03	47	M	22.5	CRC_03	81	F	24.9	Rectum	IIA
HC_04	40	M	24.7	CRC_04	63	F	29.55	Descending colon	IIA
HC_OW/OB_05	87	M	25.5	CRC_05	64	M	23.88	Rectum	IIA
HC_OW/OB_06	60	F	25.4	CRC_06	69	M	24	Rectum	I
HC_OW/OB_07	50	F	27.2	CRC_07	47	M	26.42	Rectum	IIIA
HC_OW/OB_08	75	M	28.7	CRC_08	69	M	28.04	Sigma	IIIB
HC_OW/OB_09	59	M	28.1	CRC_09	77	F	24	Sigma	IIA
HC_OW/OB_10	69	F	26.7	CRC_10	79	M	29.01	Ascending colon	IIIB
HC_11	34	F	20.6	CRC_11	39	F	26.04	Rectum	I
HC_12	35	F	23.5	CRC_12	63	F	29.4	Descending colon	IVA
HC_OW/OB_13	56	M	31.7	CRC_13	58	F	23.43	Sigma	I
HC_14	73	M	20.3	CRC_14	74	F	32.5	Ascending colon	IIA
HC_15	43	M	21.7	CRC_15	82	M	30.6	Ascending colon	IIIC
HC_16	26	M	21.8	CRC_16	79	M	28.88	Rectum	IIIB
HC_17	36	F	22.2	CRC_17	83	M	28.9	Sigma	IVA
HC_18	39	F	22.1	CRC_18	70	M	32.8	Ascending colon	IIA
HC_OW/OB_19	58	F	27.6	CRC_19	58	F	30	Sigma	IVA
HC_20	40	M	24.7	CRC_20	64	M	26.56	Sigma	IIA

Healthy Control (**HC**): n= 10 BMI≥25 (HC_OW/OB, mean ± sd = 28.7±3.5 Kg/m²) and 10 BMI<25 Kg/m² (HC_NW, mean ± sd = 22.4±1.5 Kg/m²); **CRC**: n=15 BMI≥25 (CRC_OW/OB, mean ± sd = 28.9±2.1 Kg/m²) and 5 BMI<25 (HC_NW, mean ± sd = 24.0±0.5 Kg/m²). *Tumor stage was according to the AJCC Staging System for Colon Cancer, 8th ed., 2017.

Supplementary Table S2. Differentially abundant species in CRC samples grouped by Tumor location with the associated Log2FC values and adjusted *p*-values.

	Tumor location comparisons		
	**Species**	**Log2FC**	**adjusted p-value**
R vs AC	*Akkermansia muciniphila*	23.20	4.69E-32
	Bifidobacterium longum	7.03	2.25E-05
	Bacteroides fragilis	−4.84	2.49E-03
	Fusobacterium nucleatum	−4.38	3.45E-02
	Actinomyces odontolyticus	3.13	4.23E-02
R vs S-DC	*Granulicatella adiacens*	−7.67	2.15E-03
	Bifidobacterium longum	5.33	2.15E-03
	Peptostreptococcus stomatis	−5.39	2.15E-03
	Bulleidia moorei	−4.82	2.15E-03
	Gemella haemolysans	−5.04	2.75E-03
	Parvimonas micra	−4.59	2.75E-03
	Actinomyces odontolyticus	−3.94	5.15E-03
	Alistipes onderdonkii	4.00	6.16E-03
	Bacteroides uniformis	4.62	6.16E-03
	Bacteroides fragilis	−4.02	6.95E-03
	Alistipes putredinis	3.81	7.00E-03
	Streptococcus intermedius	−4.56	8.27E-03
	Fusobacterium nucleatum	−3.69	3.94E-02
AC vs S-DC	*Akkermansia muciniphila*	−24.93	5.69E-22
	Actinomyces odontolyticus	−7.08	1.82E-04
	Alistipes putredinis	6.90	7.14E-04
	Granulicatella adiacens	−10.23	1.10E-03
	Bacteroides uniformis	6.88	3.51E-03
	Streptococcus intermedius	−7.03	3.86E-03
	Alistipes onderdonkii	5.30	1.06E-02
	Parabacteroides distasonis	4.29	1.92E-02
	Gemella haemolysans	−4.53	4.94E-02
	Bacteroides dorei	5.33	4.94E-02

AC, ascending colon; S-DC, sigma-descending colon; R, rectum.

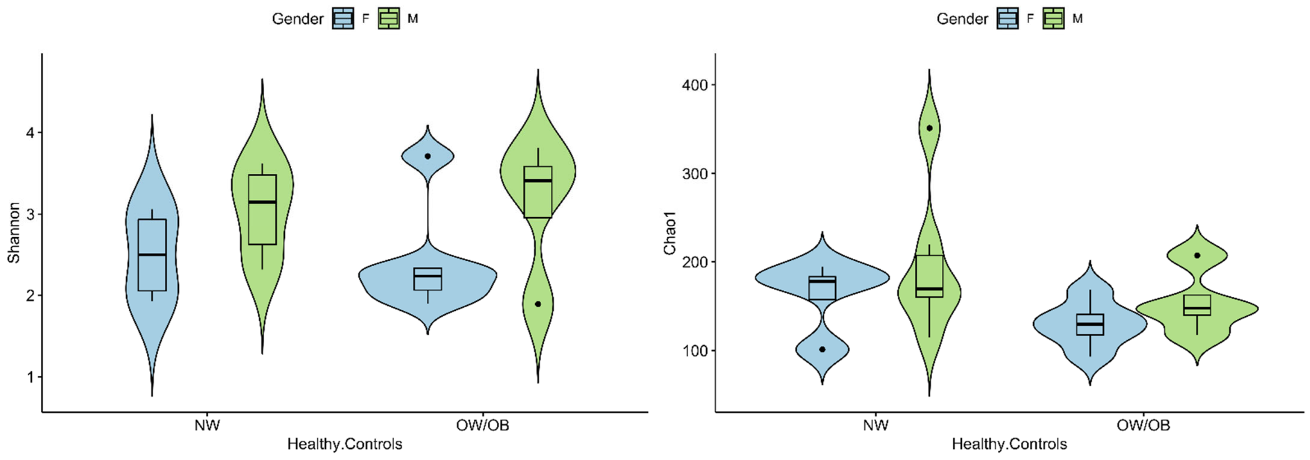

Supplementary Figure S1. Violin plot representing alpha diversity measurements. (**A**) Shannon Diversity Index and (**B**) Chao1 richness in Healthy Controls NW and OW/OB microbiomes colored by gender. Boxes span the first to third quartiles; the horizontal line inside the boxes represents the median, black dots represent all samples in each group and red dots represent outliers. No statistically significant differences have been detected in both cases.

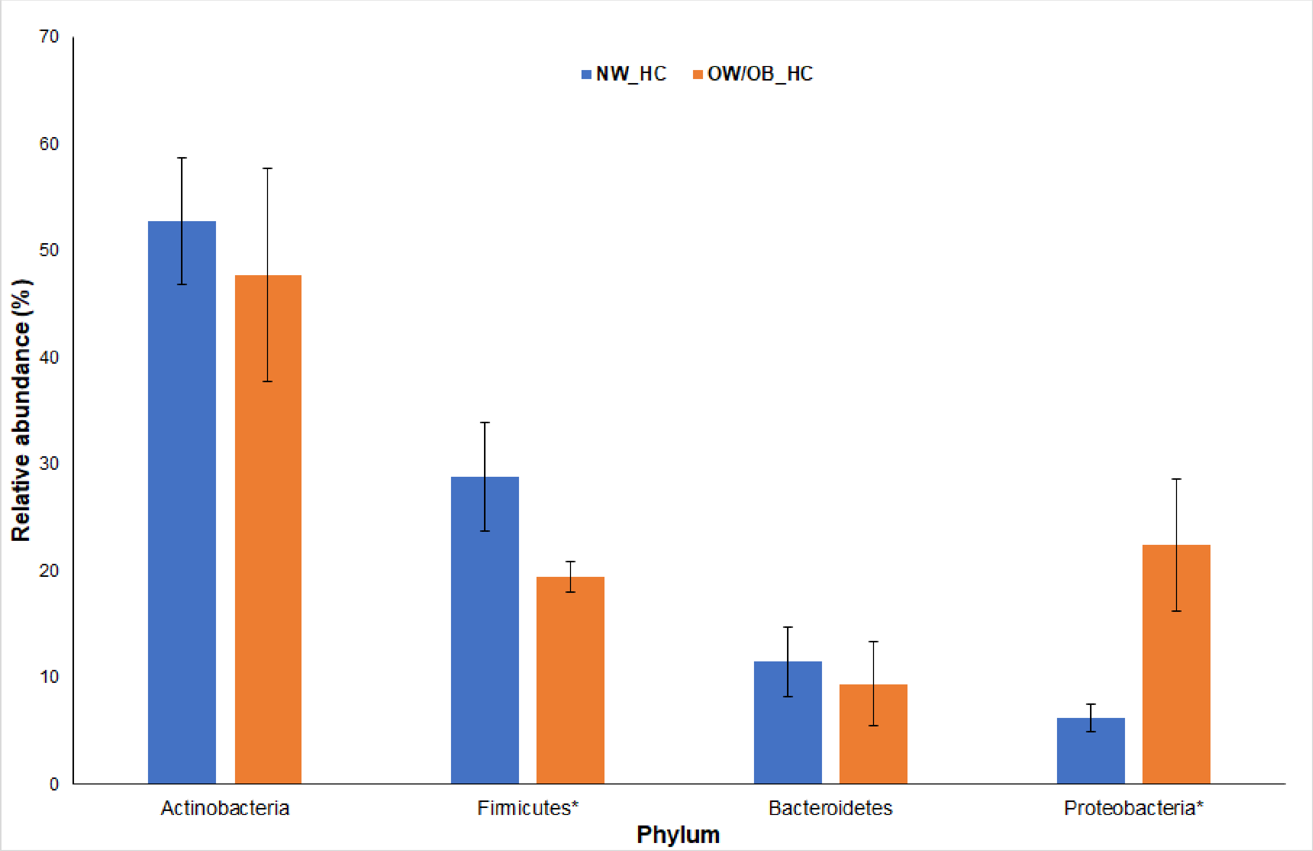

Supplementary Figure S2. Bar graph illustrating the mean ± mean standard error (error bars) of the percentage of mean relative abundance (%) of main phyla detected in OW/OB (orange) and NW healthy (blue) groups. * Statistically different relative abundance between the two groups ($p < 0.05$).

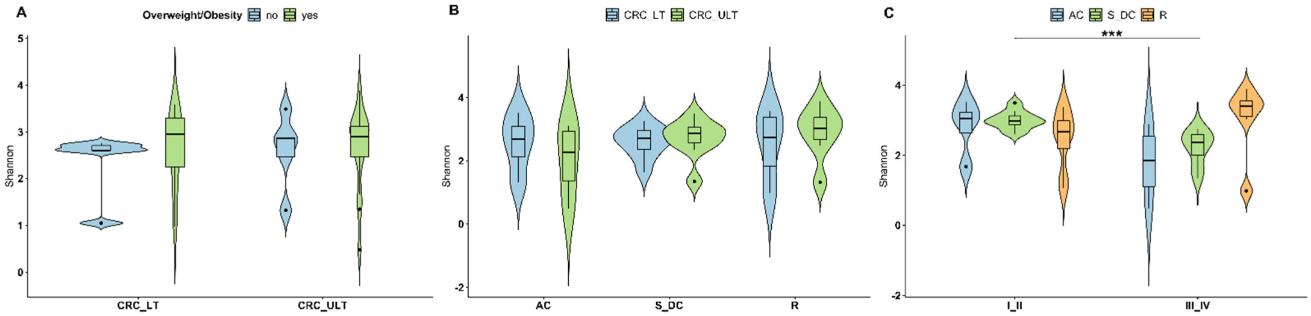

Supplementary Figure S3. Violin plot representing alpha diversity measurements in CRC microbiomes. (**A**) Shannon Diversity Index of CRC_LT and CRC_ULT samples colored by overweight/obesity comorbidity; (**B**) Shannon Diversity Index of samples grouped by the three different tumor location and colored according to CRC_LT/CRC_ULT; (**C**) Shannon Diversity Index of samples grouped by tumor stages and colored according to the tumor location. No statistically significant differences have been detected in all cases, except for the stage comparison in S-DC samples (*** < 0.005). Boxes span

the first to third quartiles; the horizontal line inside the boxes represents the median, black dots represent all samples in each group and red dots represent outliers. LT, tumor lesioned tissue; ULT, unlesioned tissue.

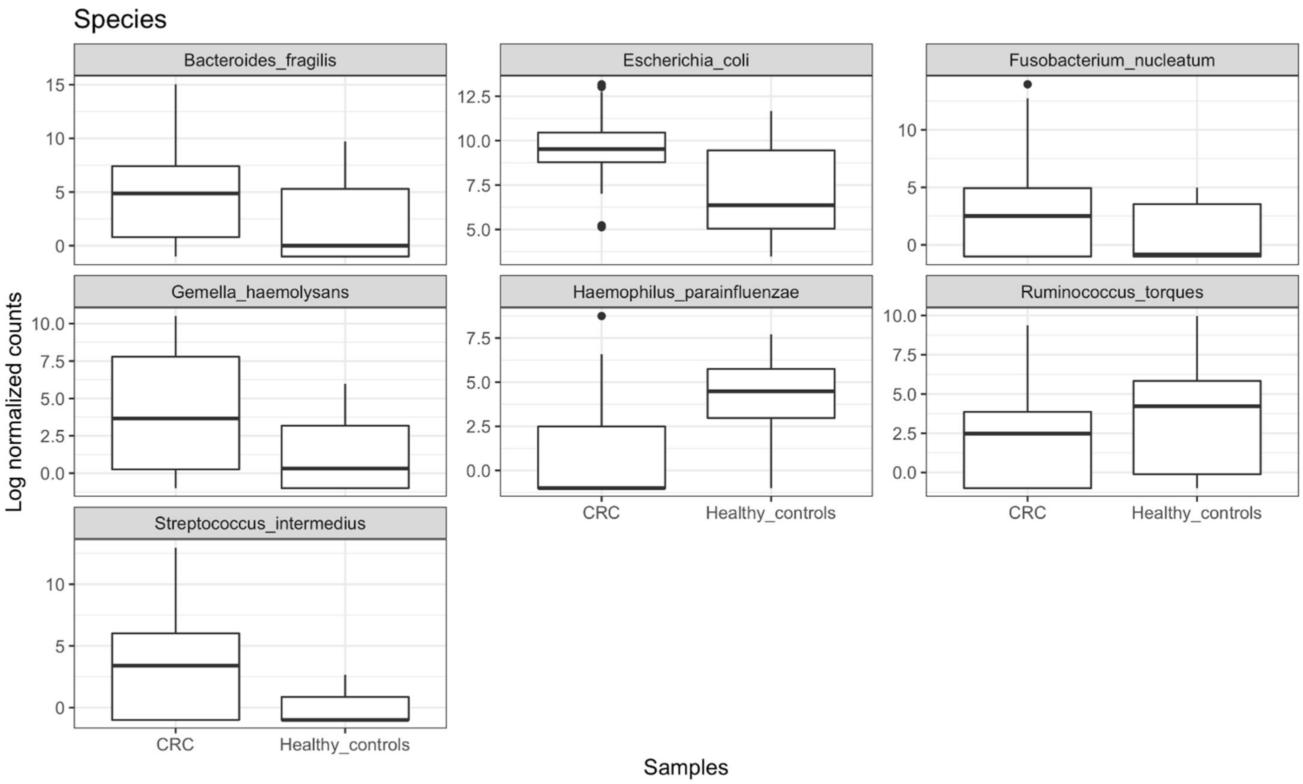

Supplementary Figure S4. Boxplots of the significant results (adjusted *p*-value < 0.05) obtained through differential abundance test between CRC and HC groups at species level. The test has been performed by applying DESeq2 Negative Binomial distribution. Lower and upper box boundaries 25th and 75th percentiles, respectively, line inside box median, lower and upper error lines 10th and 90th percentiles, respectively, filled circles data falling outside 10th and 90th percentiles.

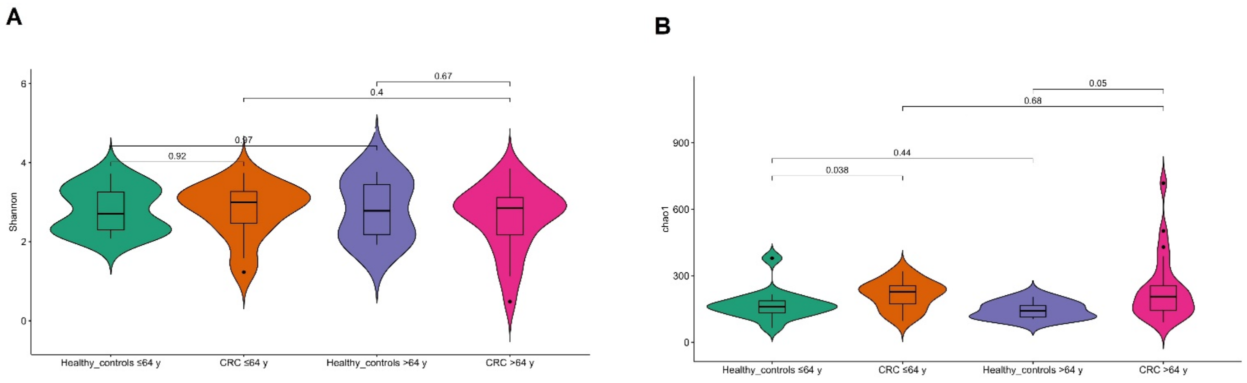

Supplementary Figure S5. Violin plot representing alpha diversity measurements. (**A**) Shannon Diversity Index and (**B**) Chao1 richness in microbiome of healthy controls and CRC divided in two groups of different age (≤ or >64 years). Boxes span the first to third quartiles; the horizontal line inside the boxes represents the median, black dots represent outliers.

No statistically significant differences have been detected in both cases, except for Chao1 in the comparison between CRC versus HC in both groups of different age.

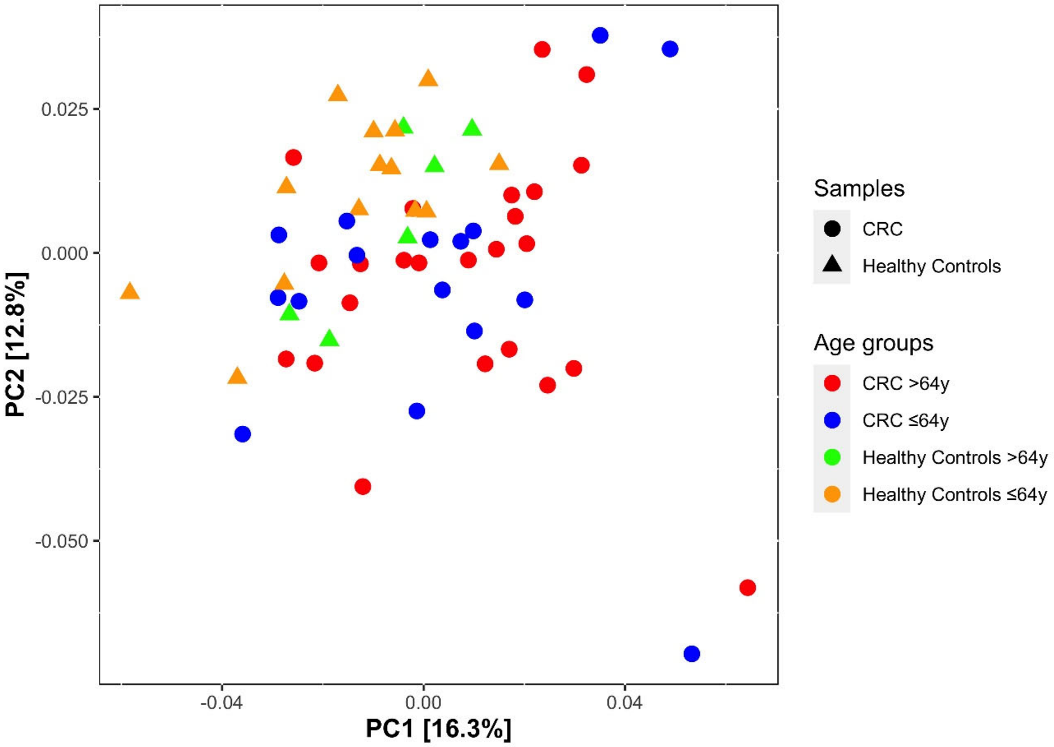

Groups	ADONIS R2	ADONIS p-value	ANOSIM R	ANOSIM p-value	Beta-disp. F value	Beta-disp. P value
**CRC/HC (≤ or >64 years)**	0,086	**0,003**	0,005	0,449	1,267	0,294

Supplementary Figure S6. Principal Coordinate Analysis (PCoA) plot of weighted UniFrac distance showing the distribution of CRC patients and healthy ontrols divided by the age cutoff (≤ or >64 years). The samples are differently colored according to the age groups and differently shaped according to CRC or healthy (healthy controls) condition. The PERMANOVA (Adonis) test suggests a significant separation of CRC patients and healthy controls considering the age range ≤ or >64 years (p = 0.003). Values obtained from Analysis of Similarity (ANOSIM) and beta-dispersion tests are also shown.

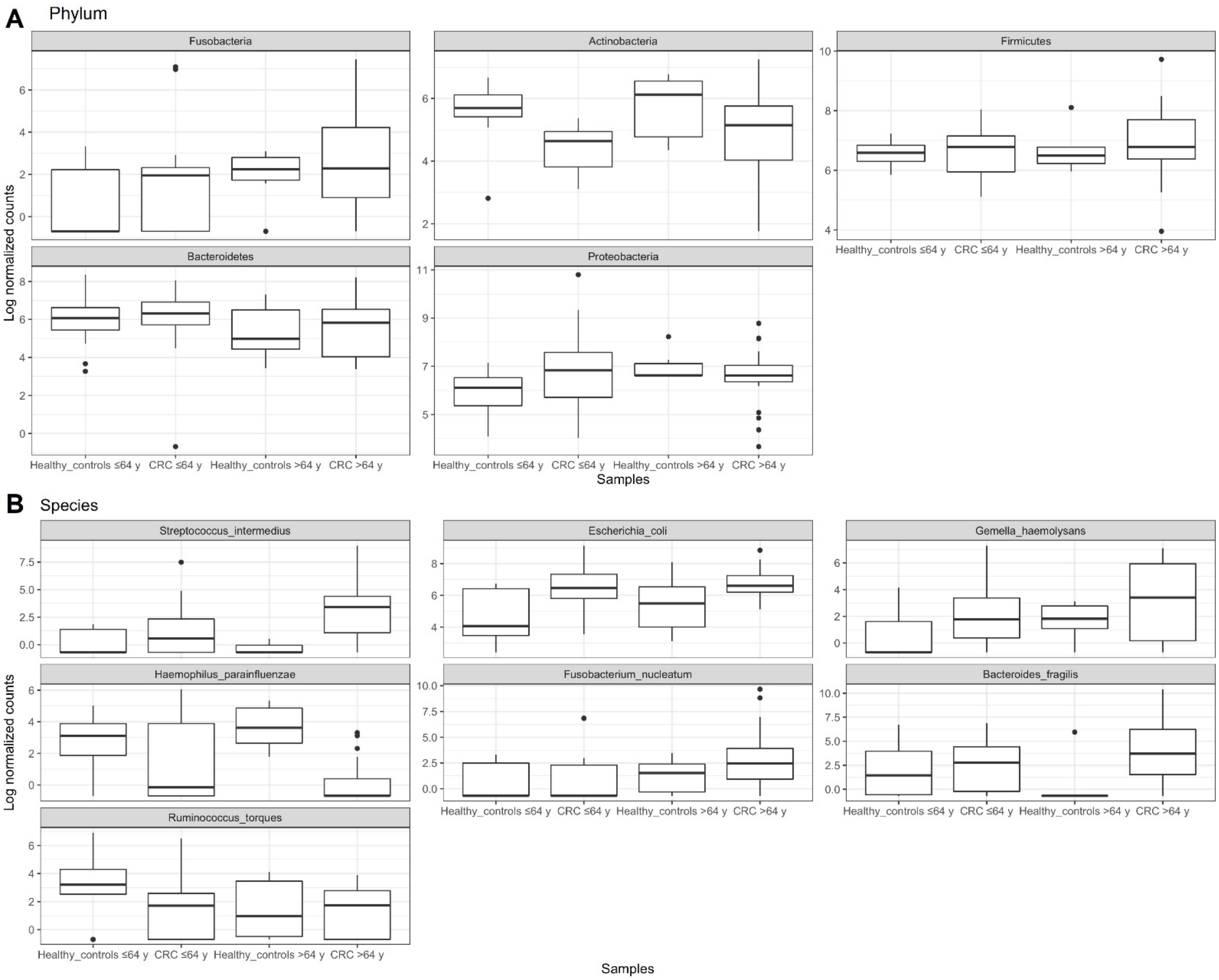

Supplementary Figure S7. Boxplots of the microbial abundance (Log normalized counts) in CRC and HC groups ≤ or >64 years at (A) phylum and (B) species levels. The statistical test has been performed by applying DESeq2 Negative Binomial distribution and modeling data by age categorization. The taxa have been grouped by taxonomy level and those having counts >1 in at least 60% of samples have been kept. Lower and upper box boundaries 25th and 75th percentiles, respectively, line inside box median, lower and upper error lines 10th and 90th percentiles, respectively, filled circles data falling outside 10th and 90th percentiles. The taxa were considered statistically significantly different at adjusted p-value < 0.05. Actinobacteria and Fusobacteria phyla (adjusted p-value < 0.001) and the species *Streptococcus intermedius, Gemella haemolysans, Fusobacterium nucleatum, Escherichia coli* (adjusted p-value < 0.001) were significantly different between CRC and HC independently from the age differences between the two groups.
